# Prevalence and associated factors of antipsychotic-induced extrapyramidal symptoms in the Tigray region

**DOI:** 10.1186/s12888-025-07645-y

**Published:** 2025-11-25

**Authors:** Etsay Hailu Gebremariam, Tesfaye Derbie Begashaw, Light Tsegay, Gebreslassie Gebreegziabhier Kindeya, Abraha Gosh Weldemariam, Welu Abadi Gebru, Haftom Desta Kahsay, Hagos Tsegabrhan

**Affiliations:** 1 Department of Psychiatry, College of Health Science, Aksum University, Axum, North Ethiopia Ethiopia; 2 Department of Psychiatry, College of Health Science, Aksum University, Mekelle, North Ethiopia Ethiopia; 3 Department of Psychiatry, College of Health Science, Aksum University, Addis Ababa, Ethiopia; 4 Department of Psychiatry, Ayder College of Health Science, Mekelle University, Mekelle, North Ethiopia Ethiopia

**Keywords:** Prevalence, Antipsychotics, Extrapyramidal symptoms

## Abstract

**Background:**

Extrapyramidal symptoms affect an average of 37%, with a maximum prevalence of 71.4%, among individuals on antipsychotics. About one in five patients experiences Parkinsonism, and more than one in ten experiences akathisia. These symptoms can significantly increase relapse rates, elevate healthcare costs (approximately $27,408), and contribute to stigmatization. In Tigray, where antipsychotic use is rising, data on Extrapyramidal symptoms are limited. This study aimed to fill this research gap to better patient care and management.

**Objectives:**

The aim of this study was to determine the prevalence of Extrapyramidal symptoms associated with antipsychotic medications. Additionally, it sought to identify factors contributing to these side effects among patients.

**Methods:**

This study was conducted across five public hospitals in the Tigray region, utilizing a comprehensive approach that included face-to-face interviews, direct observations, card reviews, and physical examinations. A total of 834 participants were selected through a multistage sampling technique to ensure representativeness, using the Extra Pyramidal Symptom Rating Scale (ESRS) for standardized evaluation. Data collected were analyzed using SPSS version 27.

**Result:**

The study findings showed that the prevalence rates of Parkinsonism, Akathisia, Dystonia, and Tardive Dyskinesia were **9.9%**,** 6.9%**,** 5.0%**,** and 2.4%**, respectively. The multivariate analysis revealed several common risk factors associated with these extrapyramidal symptoms. Both Parkinsonism and Akathisia were significantly associated with various factors, including the type of antipsychotic medication, illness relapse, comorbid medical conditions, anticholinergic medication, perceived stigma, and current khat use. Additionally, Parkinsonism was associated with the type of mental illness. Dystonia was also associated with marital status, anticholinergic medication, perceived stigma, and tobacco use. Lastly, Tardive Dyskinesia was associated with educational status and medication adherence.

**Conclusion and recommendations:**

Although Extrapyramidal symptoms were reported at lower rates than in previous studies, psychiatry professionals should routinely screen for EPS and tailor treatment to patient-specific factors. Efforts to reduce stigma around these side effects are also essential to improve patient outcomes.

## Highlights

**Research gap addressed:**

• No prior regional data existed on the prevalence or determinants of antipsychotic-induced extrapyramidal side effects (EPS) in the Tigray region.

**What was done and what was found:**

• Prevalence ranged from 2.4% (Tardive Dyskinesia) to 9.9% (Parkinsonism).

• Key associated factors included:

• Comorbid physical illnesses.

**Implications:**

• Emphasizes the need for routine EPS screening, especially among high-risk psychiatric patients.

• Supports integrated care strategies, focusing on:

• Safe medication use.

• Stigma reduction.

• Substance use interventions.

## Introduction

### Background

The utilization of antipsychotic medications is becoming more prevalent in contemporary clinical practice, reflecting a growing recognition of their role in managing various psychiatric conditions [1]. The development of antipsychotic medications began in 1952 with the release of chlorpromazine, marking a significant milestone in psychiatric treatment [2]. Two different generations of antipsychotic medications, each characterized by distinct therapeutic philosophies, emerged as a result of this revolutionary discovery. The mechanisms of action, adverse effects, and overall efficacy of first-generation antipsychotics—also known as typical antipsychotics—differ markedly from those of their second-generation counterparts, referred to as atypical antipsychotics [3, 4].Motor-related Extrapyramidal symptoms (EPS) such as tremors, rigidity, bradykinesia, and tardive dyskinesia are known to be more common with first-generation antipsychotics (FGAs) [5].On the other hand, second-generation antipsychotics (SGAs), like Aripiprazole, quetiapine, and Risperidone, were created to reduce these motor adverse effects [6]. Though SGAs are linked to a lower incidence of EPS, it’s important to note that some drugs, such as Risperidone, can still cause severe adverse effects if taken in larger dosages. In particular, dosages of Risperidone exceeding 6 mg may raise the risk of EPS, which is comparable to what can happen with FGAs [7, 8].

Extrapyramidal symptoms can manifest in two forms: acute and chronic. Conditions such as acute dystonia and akathisia are examples of acute side effects, which typically appear within hours to days and can cause significant discomfort and distress [9]. In contrast, chronic side effects take longer to develop, ranging from months to years, with one of the most concerning symptoms being tardive dyskinesia [10].Antipsychotic medications induce Extrapyramidal symptoms can have a profound impact on patients, causing severe discomfort and suffering. A person’s quality of life is greatly impacted by these effects, which frequently show up as rigidity, restlessness, tremors, and involuntary muscle spasms. Acute dystonia, for example, is characterized by abrupt and intense muscular contractions that can cause upsetting symptoms like tremors, stiff necks, and twisted facial expressions. Another example is akathisia, which causes extreme uneasiness for those who experience it due to its excessive restlessness and inability to stay motionless [11].

Then again, persistent EPS may result in longer-term issues. One of the most well-known side effects, tardive dyskinesia, developes from long-term use of antipsychotics and is characterized by repeated, involuntary movements, particularly of the mouth, tongue, and face. Patients may experience significant emotional discomfort as a result of this condition’s major impairment of social interactions and vocational functioning. Remarkably, tardive dyskinesia may be irreversible, continuing long after the drug that caused it has been stopped [12]. There are a number of risk factors that can raise the chance of developing EPS while using antipsychotic medications. Among the primary and significant risk factors are genetic predispositions, age, dosage, medication type, duration, and concurrent medications [13].

According to a comprehensive global review, the incidence rates of antipsychotic-induced Parkinsonism, akathisia, and tardive dyskinesia are approximately *20%*,* 11%*,* and 7%*, respectively [14]. When considering the broader population of patients with schizophrenia spectrum disorders on these medications, the overall prevalence of Extrapyramidal symptoms (EPS) was 42.6%. Specifically, the prevalence of Parkinsonism, akathisia, and tardive dyskinesia were *38.6%*,* 3.6%*,* and 7.9%*, respectively. Moreover, the prevalence of EPS due to the use of typical, atypical, and combination drugs was 44.4%, 51.2%, and 34.5%, respectively. Additionally, haloperidol (59.4%) and risperidone (71.4%) were associated with the greatest effects on EPS [15]. Over one-fifth of patients on atypical antipsychotics developed EPS within a year, increasing hospitalization risk. Patients with EPS had significantly higher all-cause costs ($27,408) and schizophrenia-related costs ($12,833) compared to those without EPS [16].

The anticipated rise in the use of antipsychotic medications in Tigray, underscores the critical need to raise awareness about Extrapyramidal symptoms (EPS) [15]. In low-resource settings such as ours, the range of available antipsychotic medications is limited primarily to chlorpromazine, haloperidol, and risperidone—agents that are widely used due to their affordability and inclusion in essential medicine lists. However, these drugs are well known to be associated with extrapyramidal symptoms (EPS), which can lead to medication non-adherence, relapse, and functional impairment. Despite their common use, there is a scarcity of local and regional evidence on the magnitude and determinants of EPS among patients receiving these agents in similar resource-constrained formularies. Most existing studies have been conducted in high-income settings where newer antipsychotics with lower EPS risk are more accessible, limiting their applicability to low- and middle-income countries.

Furthermore, local prescribing patterns often involve Polypharmacy, limited dose titration, and inadequate routine monitoring for EPS, partly due to workforce shortages and lack of standardized screening protocols. The sociodemographic profile of patients—characterized by high unemployment, low literacy, and delayed treatment seeking—may also modify vulnerability to EPS. Understanding the burden and risk factors of EPS in this context is therefore critical for optimizing treatment, informing local prescribing guidelines, and supporting health policy aimed at safer and more effective antipsychotic use in resource-limited settings.

Despite this, there was a notable research gap, as no studies had been conducted in Tigray to determine how common EPS actually were. To the best of our knowledge, no previous studies have comprehensively addressed this issue across different hospitals. Moreover, the current study is unique in that it is conducted in a setting with a distinct context and experience, particularly being affected by war. The relatively large sample size also strengthens the reliability and generalizability of the findings. Therefore, this study is expected to fill an important knowledge gap and provide evidence that can guide future research, policy, and practice in similar contexts. Identifying these symptoms is vital to ensure patients receive safe and effective treatment for their mental health conditions. Addressing this gap by conducting an updated study in Tigray became essential. It also highlighted the urgent need for ongoing education and support for mental health professionals in the region, to improve their ability to recognize and manage EPS effectively. Ultimately, this effort aims to enhance patient safety and treatment outcomes in the area.

## Objectives

The aim of this study was to determine the prevalence of Extrapyramidal symptoms of antipsychotic medications and associated factors among patients in Tigray general and comprehensive specialized hospitals in 2024.

## Methods and material

### Study area and period

The study was conducted in the northern part of Ethiopia, specifically in the Tigray regional state, from March 1, 2024, to April 30, 2024. The Tigray regional state is divided into six administrative zones and one special zone (Mekele). The region has a total of 40 hospitals (2 comprehensive specialized hospitals, 14 general hospitals, and 24 primary hospitals), 226 health centers, and 741 health posts.

### Source population and study population

All Patients diagnosed with mental illness, who were taking antipsychotic medications and on follow up at selected general and referral public hospitals.

### Inclusion and exclusion criteria

Patients with mental illness who had been on antipsychotic medications were included and who were severely ill, unable to give the required verbal information and who had history of primary movement disorders were excluded from the study. Participants were eligible for inclusion if they were diagnosed with bipolar disorders, were on antipsychotic treatment during the study period, and provided written informed consent to participate. Patients aged 18 years and above with a diagnosis of mental illness who had been receiving antipsychotic medications during the study period were eligible for inclusion. Individuals, who were severely ill, defined as those in an acute state of disturbance that impaired their ability to provide informed consent or participate meaningfully, were excluded.

### Sample size determination

The sample size was calculated using the single population proportion formula, based on the following assumptions: a prevalence (p) of 56% (from a study conducted at Amanuel Mental Specialized Hospital), a 95% confidence interval, a 5% margin of error, and a 10% non-response rate. The initial calculated sample size was 379. After accounting for the 10% non-response rate, the adjusted sample size increased to 417. To consider potential design effects, this number was multiplied by a design effect of 2, resulting in a final sample size of 834 participants (417 × 2 = 834).

### Sample size calculation details

The sample size was calculated using the single population proportion formula with the following assumptions:

Prevalence (p) = 56%, obtained from a study at Amanuel Mental Specialized Hospital

Confidence level = 95% (z = 1.96)

Margin of error = 5%

Non-response rate = 10%

The formula used:

$$\:n=\:\frac{({Z\alpha\:/2)}^{2}\times\:p(1-p)}{{d}^{2}}$$

Where:

Z = 1.96

*p* = 0.56p

d = 0.05

Calculating:

Then, n = $$\:\frac{\left(1.96\right)2\left[0.56\text{*}\left(1-0.56\right)\right]}{\left(0.05\right)2}=379$$.

Adding 10% for non-response:

379+(0.10 × 379) = 417,379 + (0.10 × 379) = 417,379+(0.10 × 379) = 417.

Applying a design effect of 2:

417 × 2 = 834,417 × 2 = 834,417 × 2 = 834.

Sample sizes for associated factors

**Table:** 

Variables	Assumption	Sample size
Age(≥ 45)	OR = 9, *P* = 33.2, Ratio = 8:3, power = 80%, CI = 95%	84
Substance (alcohol use)	OR = 4.71, *P* = 34.5%, Ratio = 12:1, power = 80%, CI = 95%	305
Dose(chlorpromazine equivalent dose ≥ 600 mg/day)	OR = 5.6, *P* = 19.8%, Ratio1:1, power = 80, CI = 95%	162

Since the sample size based on the single population proportion (834) was larger than the sample sizes calculated for associated factors (326, 303, and 252), the final sample size of 834 was deemed appropriate for the study.

### Sampling method and technique

A multi-stage sampling technique was employed for this study. In the first stage, we randomly selected five zones from the Tigray Regional State using a lottery method. Next, from each zonal administration, we identified and selected weredas (districts). From each district, we screened for general and tertiary hospitals that provide mental health services. After identifying suitable hospitals, we randomly selected one hospital from each district using a computer-generated method. Finally, we applied proportional allocation to determine the sample sizes for each selected hospital, ensuring that our sampling method accurately reflected the distribution of mental health services across the region. Accordingly, we studied 44 patients from Kahasy Abera General Hospital, 70 from Sihul General Hospital, 83 from St. Mary General Hospital, 529 from Ayder Comprehensive Specialized Hospital, and 108 from Lemlem Karl Hospital. For the study subjects, systematic random sampling was then employed. To determine Sampling interval (k) by dividing total study population (1702) during 4 weeks data collection period by a total sample size (834). K = N/*n* = 1702/834 = 2.04 ~ 2.Therefore, Participants was selected from patients every 2 intervals and the first participant was selected by lottery method, and then continued every 2.

### Dependent variable

#### Extrapyramidal symptoms (EPS)

absent or present.

### Independent variables

#### Socio-demographic factors

Age, Sex, Educational level, marital status, Residence, **and** Occupation.

#### Clinical factors

Diagnosis of health condition, Duration of treatment, Co morbid psychiatric diagnosis, chronic medical illness, **and** the family history of primary movement disorder.

#### Medication-related factors

Class of antipsychotics, Dose of antipsychotics, Concomitant medication use, Medication for movement disorders, Medication non-adherence, **and** Stigma.

#### Substance-related factors/behavioral factors

Alcohol consumption, Khat chewing, Cigarette smoking, **and** physical exercise.

### Operational definitions

#### *The presence versus absence of parkinsonism, akathisia, dystonia and tardive dyskinesia*

was determined based on the Extrapyramidal Symptom Rating Scale (ESRS), in which each type of extrapyramidal symptom is defined as present if the patient has at least a score of one for each extrapyramidal symptom [17].

#### Current substance use

using at least one of a specific substance for nonmedical purpose within the last 3 months.

#### Ever use of substance

using at least one of any specific substance for the nonmedical purpose at least once in a lifetime.

#### Presence of chronic diseases

when subjects have at least one or more chronic disease Such as Diabetes, HTN, Heart disease, HIV, Tuberculosis, Asthma and Cancer.

#### Medication non-adherence

The MMAS-4 scale is utilized to evaluate adherence to antipsychotic medication. According to this scale, a score of 0 or 1 signifies poor adherence, indicating that the patient is not effectively following their medication regimen. A score of 2 or 3 represents moderate adherence, suggesting that the patient occasionally adheres to their medication plan but may miss doses. In contrast, a score of 4 reflects good adherence, demonstrating that the patient consistently takes their medication as prescribed [18].

### Data collection tools

Data was collected using structured questionnaire which has eight sections: a socio-demographic questionnaire, substance-related factors and clinical related questions. Medication-related factors were obtained from the patient medical record. Medication non-adherence was measured by the Morisky medication adherence scale-4. Extra pyramidal Symptom Rating Scale (ESRS) was used to assess the presence of antipsychotic-induced movement disorders, WHO-QAL BREEF, Jacobs perceived stigma scale.

Extrapyramidal symptoms were assessed using the **Extrapyramidal Symptom Rating Scale (ESRS)**, originally developed by Chouinard and Ross-Chouinard (1980). The instrument has been appropriately cited in the reference list [19].

The ESRS was administered by trained examiners who were psychiatry professionals holding at least a bachelor’s degree in psychiatry, and the majority were master’s-level specialists. All examiners received structured training on the administration and scoring of the ESRS prior to the commencement of data collection. The training covered item definitions, rating procedures, and interpretation of scores to ensure consistency in administration.

To enhance reliability, a pilot exercise was conducted on a small group of participants not included in the final sample. During this phase, inter-rater reliability was evaluated, and discrepancies in scoring were reviewed and resolved through consensus discussions. In addition, ongoing supervision was provided during data collection to monitor adherence to standardized procedures. These steps were undertaken to ensure both the accuracy and consistency of the ESRS assessments throughout the study.

The Extra pyramidal Symptom Rating Scale (ESRS) is developed to assess four types of drug-induced movement disorders: Parkinsonism, Akathisia, dystonia, and tardive dyskinesia (TD). The ESRS consists of four subscales and four CGIS scales: **The Extrapyramidal Symptom Rating Scale (ESRS)** is a standardized and validated instrument designed to assess the presence and severity of four major types of drug-induced movement disorders: **drug-induced parkinsonism**,** dystonia**,** akathisia**,** and tardive dyskinesia**. The ESRS has demonstrated **high sensitivity and specificity**, particularly when administered by trained clinicians. In previous psychometric evaluations, the ESRS showed sensitivity values ranging from **78% to 92%** and specificity between **80% and 95%**, depending on the EPS subtype and the reference standards used (e.g., clinical neurological examination or expert consensus).

The ESRS offers comprehensive coverage of both subjective and objective symptom domains, making it an effective tool for early detection, monitoring, and clinical decision-making in patients receiving antipsychotic medications. Its structure allows for the separate rating of each EPS subtype, providing nuanced data for epidemiological and clinical studies.

In the **Ethiopian context**, although large-scale validation studies are limited, the ESRS has been used in multiple psychiatric facilities, including Amanuel Mental Specialized Hospital, and is considered appropriate for assessing EPS in patients on antipsychotic therapy. The scale’s feasibility, low resource requirement, and ease of administration make it particularly suitable for use in resource-limited settings like Ethiopia, where access to advanced diagnostic tools may be constrained. Furthermore, its structured format supports standardized assessments across diverse healthcare providers and facilities.

i. **A Questionnaire of EPS or DIMD**: For the subjective examination scoring is on a 4-point scale (0 = absent; 1 = Mild, 2 = Moderate, 3 = Severe).
ii. **An examination of Parkinsonism and Akathisia**: The Parkinsonism score, ranging from 0 to 96 (16 items).The score for Akathisia (0–6) is based on the combined score of subjective Akathisia (item 6 of the questionnaire) and objective Akathisia (item 7 of the Parkinsonism/Akathisia objective examination).
iii. **An examination of dystonia**: The score for dystonia ranges from 0 to 60 (10 items), and is formed by including both acute and chronic dystonia, based on the dystonia examination.
iv. **An examination of dyskinesia**: Score for TD, ranging from 0 to 42, is based on the sum of all 7 items in the TD objective examination.
v. **To VIII) clinical global impression severity (CGI-S) scales of tardive dyskinesia**,** Parkinsonism**,** dystonia and Akathisia**: are rated according to results of the subjective questionnaire, examination subscales, and the evaluator’s clinical experience by applying an 8 point rating (0: absent; 1: borderline; 2: very mild; 3: mild; 4: moderate; 5: moderately severe; 6: marked; 7: severe; 8: extremely severe). The 4 CGI-S’s are analyzed as separate items.

### Data collection techniques

Data were collected through a comprehensive approach that included face-to-face, interviewer-administered techniques, medical chart reviews, direct observations, and examinations of the patients. This multifaceted data collection method ensured a thorough understanding of the study participants.

### Data quality control

A pre-tested questionnaire was used to collect information. A questionnaire was translated into Amharic then translated back to English to check for consistency and understandability of the tool. Training was given for data collectors. The questionnaire was checked for its reliability, clarity, and simplicity. During data collection, the questionnaire was checked for its completeness on daily basis by data collectors and supervisors.

### Data processing and analysis

The data was edited, cleaned, coded and entered into Epi-data 3.1 version and analyzed by using SPSS 27 version. Bivariate and multivariate logistic regression analysis was used to identify the associated factors. The p-value less than 0.05 were considered as statistically significant. The strength of the association was presented by odds ratio with 95% C.I and Hosmer-Lemeshow goodness was used to check model fits.

### Assumptions and potential limitations

The analysis conducted in this study was grounded on several fundamental assumptions that are essential for ensuring the validity and reliability of the findings. First, it was presumed that the measurement tools used to assess various factors—such as medication adherence, side effects, and stigma—maintained their original psychometric properties even after translation into Amharic. To support this, the tools underwent rigorous pre-testing and validation procedures, including forward and backward translation, to ensure that they accurately captured the intended constructs within the cultural context. Second, the study relied on the assumption that participants responded honestly and accurately to the self-report questionnaires. While self-report methods are practical and commonly used, they are inherently vulnerable to biases, such as social desirability bias—where participants might overstate their adherence to appear compliant—and recall bias, which can lead to inaccuracies in reporting past behaviors or experiences. Third, the study assumed that the data obtained from medical records were complete, accurate, and reflective of the actual medication history of the participants. However, record-keeping practices can vary between facilities and practitioners, potentially leading to missing or erroneous data that might influence the results. Lastly, the statistical analysis, particularly the regression modeling, relied on several assumptions. It was assumed that predictor variables did not exhibit multicollinearity—that is, they were not highly correlated with each other—which could distort the model’s estimates. Additionally, the relationship between continuous predictors and the outcome was assumed to be linear on the logit scale, observations were presumed to be independent of each other, and the sample size was considered sufficient to produce reliable estimates. To verify these assumptions, comprehensive diagnostic checks—including assessments of multicollinearity, outliers, and data normality—were performed.

Despite these carefully considered assumptions, several limitations could potentially impact the robustness of the study’s conclusions. Response bias remains a concern, as participants might have consciously or unconsciously exaggerated their medication adherence or minimized side effects to present themselves in a more favorable light, thus skewing the results. In addition, inaccuracies or incompleteness in medical records could have led to misclassification or underestimation of medication-related data, affecting the study’s accuracy. Furthermore, violations of the statistical assumptions—such as the presence of outliers that disproportionately influence the model, non-normal distribution of certain variables, or high correlation among predictors—could have compromised the validity of the regression estimates. Although diagnostic procedures were employed to detect and address these issues, residual violations might still have introduced bias or reduced the precision of the findings. Overall, while steps were taken to minimize these limitations, acknowledging their potential influence is crucial for interpreting the results with appropriate caution.

### Ethics approval and consent to participate

Ethical clearance for the study was obtained from the Institutional Board Research Review Committee of the College of Health Sciences at Aksum University (**IRB Number: 28/11/2019).** Permission letters were also obtained from the respective city administrations. **All study participants were 18 years of age or older.** Informed consent was obtained from all participants, and the confidentiality of the collected information was strictly maintained. Participants who screened positive for extrapyramidal symptoms (EPS) were referred to appropriate healthcare providers for further evaluation and management. The study was conducted according to the Declaration of Helsinki.

## Results

### Socio-demographic, clinical, and medication characteristics of respondents

Out of a total of 834 participants, 817 patients were enrolled and actively participated in the study, resulting in a response rate of 97.9%.Among the respondents more than half of the participants were female 443(54.2). The mean age of the participants was 41 ± 14.31 years, which ranged from 18 to 85. The majority of the respondents, 654 (80%), identified as followers of the Orthodox religion, and Tigrean in ethinicity 785 (96.1) (Table 1).

**Table 1:** Socio-demographic, clinical, and medication characteristics of study participants at public general and referral hospitals, Tigray, Ethiopia (*n* = 817), 2024

Variable	Category	Frequency (*n*)	Percent (%)
Sex	Male	374	45.8
	Female	443	54.2
Age (years)	18–20	20	2.4
	21–40	428	52.4
	41–60	271	33.2
	> 60	98	12.0
Religion	Orthodox	654	80.0
	Muslim	163	20.0
Ethnicity	Tigray	785	96.1
	Otherᵃ	32	3.9
Marital status	Married	422	51.7
	Single	256	31.3
	Divorced	92	11.3
	Widowed	47	5.8
Educational level	Cannot read/write	221	27.1
	Can read/write	176	21.5
	Grades 1–8	137	16.8
	Grades 9–12	110	13.5
	College and above	173	21.2
Occupational status	Housewife/Unemployed	158	19.3
	Farmer	283	34.6
	Merchant	147	18.0
	Government employee	146	17.9
	Student	55	6.7
	Otherᵇ	28	3.4
Monthly income	Under poverty line	709	86.8
	Above poverty line	108	13.2
Diagnosis	Schizophrenia	536	65.6
	Other psychotic disorders	150	18.4
	Bipolar disorder	46	5.6
	Major depressive disorder	85	10.4
Relapse rate	Yes	246	30.1
	No	571	69.9
Duration of treatment	< 1 year	106	13.0
	1–5 years	503	61.6
	> 5 years	208	25.5
Comorbid psychiatric diagnosis	Yes	156	19.1
	No	661	80.9
Comorbid medical diagnosis	Yes	35	4.3
	No	782	95.7
Family history of movement disorder	Yes	38	4.7
	No	779	95.3
Ever used khat	Yes	90	11.0
	No	727	89.0
Currently using khat	Yes	10	1.2
	No	807	98.8
Ever used alcohol	Yes	574	70.3
	No	243	29.7
Currently using alcohol	Yes	107	13.1
	No	710	86.9
Ever smoked cigarettes	Yes	35	4.3
	No	782	95.7
Currently smoking cigarettes	Yes	6	0.7
	No	811	99.3
Ever used hashish	Yes	12	1.5
	No	805	98.5
Currently using hashish	Yes	2	0.2
	No	815	99.8
Ever used any substance	Yes	622	76.1
	No	195	23.9
Currently using any substance	Yes	107	13.1
	No	710	86.9
Class of antipsychotics	Chlorpromazine	290	35.5
	Risperidone	160	19.6
	Haloperidol	316	38.7
	Otherᶜ	51	6.2
Other medications (non-antipsychotics)	Yes	178	21.8
	No	639	78.2
Anticholinergic medication use	Yes	208	25.5
	No	609	74.5
Medication non-adherence	Yes	312	38.2
	No	505	61.8
Perceived stigma	Yes	339	41.5
	No	478	58.5

ᵃ Other ethnicities include: Amhara (*n* = 19), Afar (*n* = 7), Oromo (*n* = 6)

ᵇ Other jobs include: retired (*n* = 9), private sector (*n* = 14), driver (*n* = 5)

ᶜ Other antipsychotics include: Olanzapine, Trifluoperazine, Fluphenazine, Thioridazine

Among the study participants, most of the respondents 536 (65.6%) had the diagnosis of schizophrenia. More than half of study participants 503 (61.6%) had received treatment within the range of 1–5 years. Among the study participants, the majority, 574 (70.3%), reported having used alcohol at least once in their lifetime, while only 107 (13.1%) of the respondents were current users. In this study, 1.5% of the respondents used hashish once in their life time (Table 1).

In general, 622 respondents (76.1%) reported being lifetime users of any substance, while 107 respondents (13.1%) were identified as current users. Among the study participants, 316 (38.7%) respondents were taking Haloperidol, and 178 (21.8%) of the respondents were taking concominent medications. More than one-third of the study participants, 312 (38.2%), reported that they were not taking their medication effectively. Additionally, over half of the participants indicated experiencing stigma related to their condition (Table 1).

### Prevalence of extrapyramidal symptoms of antipsychotics (EPS)

**Fig. 1:**
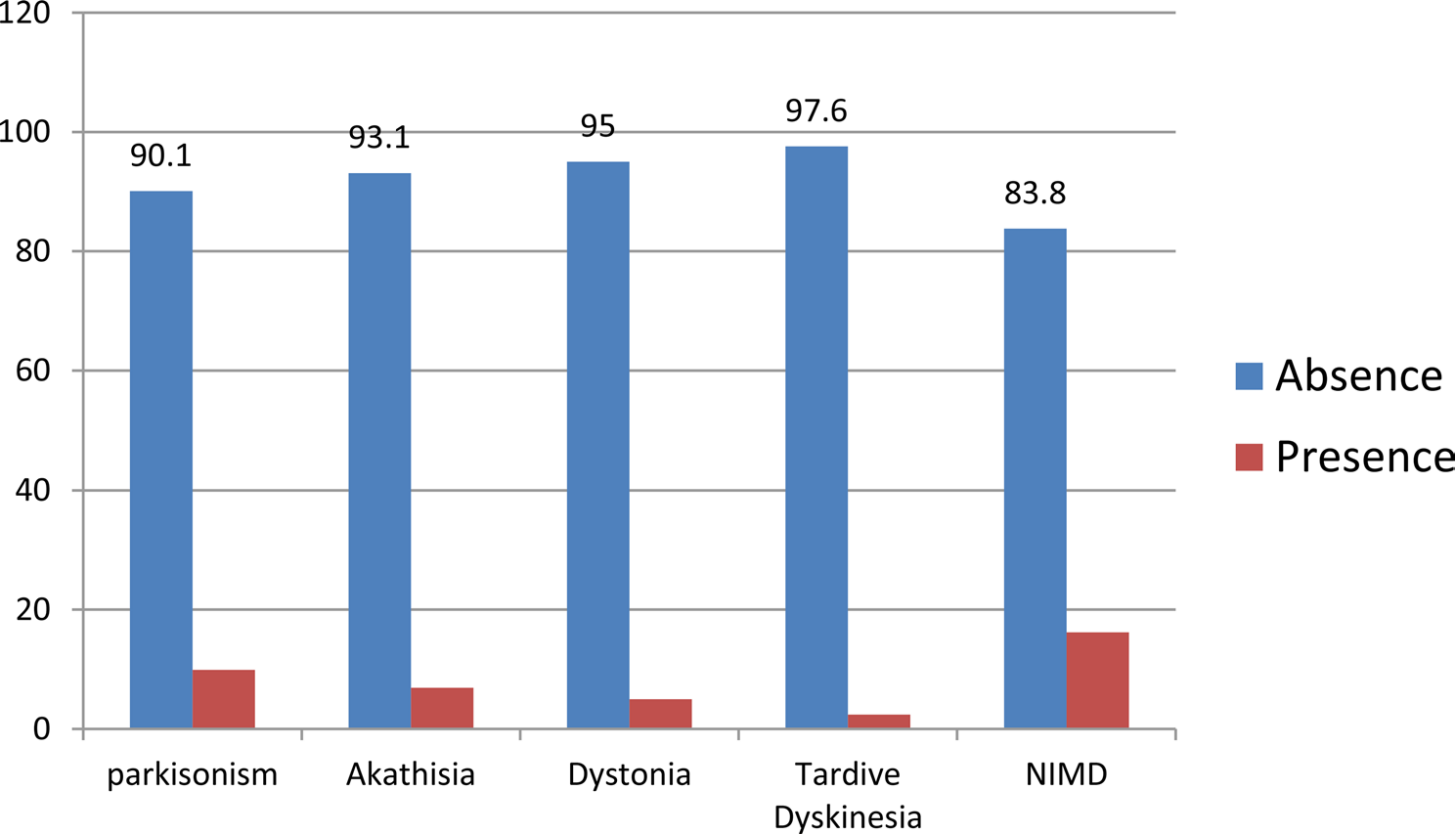
Prevalence of Neuroleptic induced movement disorder among study participants at general and referral public hospitals in Tigray, Ethiopia, 2024/2024(*n* = 817)

The prevalence of each type of Extrapyramidal symptoms (EPS) among the study participants was as follows: Parkinsonism was reported by 81 individuals (9.9%), akathisia was observed in 56 participants (6.9%), dystonia was present in 41 individuals (5.0%), and tardive dyskinesia was reported in 20 participants (2.4%) (Fig. 1).

### Factors associated with acute and chronic extra-pyramidal symptoms

The result of multivariate analysis shows that neuroleptic-induced Parkinsonism was significantly associated with types of mental illness, types of anti-psychotics, illness relapse, and comorbid medical illness, medication for neuroleptic induced malignant disorder, comorbid medical illness, perceived stigma and current use of khat. The odds of developing neuroleptic induced Parkinsonism among clients who had a diagnosis of Bipolar disorder were 3 times **(AOR = 3.13**,** 95%CI: 1.05**,** 9.25)** higher as compared to clients with a diagnosis of major depressive disorder. Clients having comorbid medical illness were 3 times **(AOR = 3.16**,** 95%CI: 1.32**,** 7.54)** more likely to develop neuroleptic induced Parkinsonism as compared to those clients without comorbid medical illness (Table 2).

**Table 2:** Factors associated with antipsychotic-induced akathisia among patients at public hospitals in Tigray, Ethiopia, 2024 (*n* = 817)

Variable	Category	Akathisia		COR (95% CI)	AOR (95% CI)
		(Yes)	(No)		
Educational status	Unable to read/write	10	211	0.49 (0.22–1.14)	–
	Able to read/write	13	163	0.84 (0.38–1.82)	–
	Grades 1–8	10	127	0.82 (0.36–1.91)	–
	Grades 9–12	8	102	0.82 (0.33–2.01)	–
	College and above	15	158	(Ref)	–
Illness relapse	Yes	23	223	1.68 (0.96–2.92)	2.17 (1.31–3.60)**
	No	33	538	(Ref)	(Ref)
Type of antipsychotic	Chlorpromazine	10	280	(Ref)	(Ref)
	Risperidone	15	145	2.89 (1.27–6.60)*	2.00 (0.89–4.47)
	Haloperidol	27	289	2.61 (1.24–5.50)*	2.64 (1.33–5.22)**
	Otherᵃ	4	47	2.38 (0.71–7.91)	3.42 (1.32–8.82)*
Medication for NIMD	Yes	24	185	2.35 (1.35–4.09)**	2.06 (1.23–3.45)**
	No	32	576	(Ref)	(Ref)
Medication non-adherence	Yes	30	282	1.96 (1.13–3.38)*	–
	No	26	479	(Ref)	–
Perceived stigma	Yes	39	300	3.52 (1.95–6.34)***	3.52 (2.08–5.96)***
	No	17	461	(Ref)	(Ref)
Ever used khat	Yes	15	75	2.11 (1.04–4.24)*	–
	No	66	661	(Ref)	–
Current khat use	Yes	4	6	9.67 (2.64–35.37)**	6.99 (1.74–27.95)**
	No	52	755	(Ref)	(Ref)
Current alcohol use	Yes	12	95	1.91 (0.97–3.75)	–
	No	44	666	(Ref)	–
Ever used tobacco	Yes	8	27	4.53 (1.95–10.50)***	–
	No	48	734	(Ref)	–

COR, crude odds ratio; AOR, adjusted odds ratio; NIMD, neuroleptic-induced movement disorder; Ref., reference category

*P* < 0.05; *P* < 0.01; *P* < 0.001. Hosmer–Lemeshow test = 0.064

ᵃOther antipsychotics include olanzapine, trifluoperazine, fluphenazine, and thioridazine

The odds **of developing neuroleptic induced Parkinsonism** among clients who had illness relapse were 2 times **(AOR = 2.11**,** 95%CI: 1.26**,** 3.54)** higher as compared to married clients. Clients taking Haloperidol were 2 times more likely to develop neuroleptic-induced Parkinsonism with an adjusted odds ratio (AOR) of 2.30 and a 95% confidence interval (CI) of 1.14 to 4.65. Additionally, those on other antipsychotic medications were 2.86 times more likely to develop neuroleptic induced Parkinsonism compared to those taking Chlorpromazine, with an AOR of 2.86 and a 95% CI of 1.08 to 7.55. Clients taking anticholinergic medication were 2 times (AOR = 2.15, 95% CI: 1.27, 3.65) more likely to develop neuroleptic induced Parkinsonism compared to their counterparts. Clients who have perceived stigma were 3.47 times (AOR = 3.47, 95% CI: 2.03, 5.93) more likely to develop neuroleptic induced Parkinsonism than clients without perceived stigma. The odds of developing neuroleptic induced Parkinsonism among clients who are current khat users were 8 times (AOR = 8.18, 95% CI: 2.01, 33.25) higher than among clients who had not taken khat (Table 2).

The result of multivariate analysis **shows that Akathisia** was significantly associated with illness relapse, anticholinergic medication, types of ant-psychosis, perceived Stigma and khat use. The odds of developing Akathisia among illness relapse clients were 2.17 times (AOR = 2.17, 95%CI: 1.31, 3.60) higher as compared to no history of illness relapse. Clients taking haloperidol were 2.64 times more likely to develop akathisia compared to those taking chlorpromazine (Adjusted Odds Ratio [AOR] = 2.64, 95% Confidence Interval [CI]: 1.33–5.22). Additionally, clients using other types of antipsychotics were 3.42 times more likely to develop akathisia (AOR = 3.42, 95% CI: 1.32–8.82). Clients who are taking anticholinergic medication were 2.06 times (AOR = 2.06, 95% CI: 1.23, 3.45) more likely to develop akathisia compared to their counterparts. The odds of developing akathisia among clients who have perceived stigma were 3.52 times (AOR = 3.52, 95% CI: 2.08, 5.96) more likely than among clients without perceived stigma. The odds of developing akathisia among clients who are current khat users were 6.99 times (AOR = 6.99, 95% CI: 1.74, 27.95) more likely to develop akathisia than among clients who had not taken khat (Table 2).

The results of the multivariate **analysis show that dystonia** was significantly associated with marital status, anticholinergic medication, perceived stigma, and tobacco use. The odds of developing dystonia among divorced clients were 3.98 times (AOR = 3.98, 95% CI: 1.67, 9.49) higher compared to married clients. Clients who are taking anticholinergic medication were 3.41 times (AOR = 3.41, 95% CI: 1.76, 6.59) more likely to develop dystonia compared to those not taking anticholinergics. The odds of developing dystonia among clients who have perceived stigma were 2.8 times (AOR = 2.8, 95% CI: 1.40, 5.59) more likely to develop dystonia than clients without perceived stigma. The odds of developing dystonia among clients who are current khat users were 11 times (AOR = 11.02, 95% CI: 1.67, 72.74) more likely to develop dystonia than clients who had not taken tobacco (Table 3).

**Table 3:** Factors associated with antipsychotic-induced dystonia and dyskinesia among patients taking antipsychotics in general and referral Hospitals, Tigray, Ethiopia, 2024 (*n* = 817)

Variable	Category	Dystonia		COR	AOR
		Yes	No		
Marital status	Married	13	409	(Ref)	(Ref)
	Single	15	241	1.95(0.92,4.18)	1.38(0.62,3.08)
	Divorced	11	81	4.27(1.84,9.87)**	3.98(1.67,9.49)**
	Widowed	2	45	1.39(0.31,6.39)	2.92(0.76,11.19)
Types of anti-psychotics	Chlorpromazine	8	282	(Ref)	(Ref)
	Risperidone	8	152	1.85(0.68,5.04)	
	Haloperidol	24	292	2.89(1.28,6.55)*	
	Other	1	50	0.71(0.08,5.76)	
Medication for NIMD	Yes	22	186	3.6571.94,6.93)***	3.41(1.76,6.59)***
	No	19	590	(Ref)	(Ref)
Drug none adherence	Yes	25	287	2.66(1.39,5.07)**	
	No	16	489	(Ref)	
Perceived Stigma	Yes	27	312	2.87(1.48,5.55)**	2.80(1.40,5.59)**
	No	14	464	(Ref)	(Ref)
Current use of tobacco	Yes	2	4	9.89(1.75,55,69)**	11.02(1.67,72.74)*
	No	39	772	(Ref)	(Ref)
		**Dyskinesia**			
		**Yes**	**No**		
Marital status	Married	6	416	(Ref)	
	Single	5	251	1.38(0.41,4.57)	
	Divorced	7	85	5.71(1.87,17.41)**	
	Widowed	2	45	3.08(0.60,15.72)	
Illness relapse	Yes	12	234	3.60(1.45,8.94)**	
	No	8	563	(Ref)	
Educational status	Unable to write and read	6	215	2.38(0.47,11.97)	2.52(0.49,12.76)
	Able to write and read	1	175	0.48(04,5.43)	0.59(0.05,6.62)
	1–8	10	127	6.73(1.45,31.26)*	9.97(2.09,47.63)**
	9–12	1	109	0.78(0.07,8.75)	0.98(0.08,11.14)
	College and above	2	171	(Ref)	(Ref)
Anti-psychotics Types	Chlorpromazine	4	286	(Ref)	
	Risperidone	1	159	0.45(0.05,4.05)	
	Haloperidol	13	303	3.06(0.98,9.51)	
	Others	2	49	2.91(0.52,16036)	
Medication for NIMD	Yes	9	199	2.45(1.00,6.02)*	
	No	11	598	(Ref)	
Medication none adherence	Yes	17	295	9.64(2.80,33.18)***	1186(3.36,41.78)***
	No	3	502	(Ref)	(Ref)
Perceived Stigma	Yes	15	324	4.38(1.57,12.16)**	
	No	5	473	(Ref)	
Ever use of tobacco	Yes	3	32	4.21(1.17,15.13)*	
	No	17	765	(Ref)	

COR, crude odds ratio; AOR, adjusted odds ratio; Ref., reference category

*P* < 0.05; *P* < 0.01; *P* < 0.001. Hosmer–Lemeshow test = 0.053

The results of the multivariate **analysis show that dyskinesia** was significantly associated with educational status and medication non-adherence. The odds of developing dyskinesia among clients in grade one to eight were 9.97 times higher (AOR = 9.97, 95% CI: 2.09, 47.63) compared to those who achieved education up to college and above. Clients who were not adherent to their medication were 11.86 times more likely to develop dyskinesia (AOR = 11.86, 95% CI: 3.36, 41.78) compared to those who were taking their medications effectively (Table 3).

The result of multivariate analysis shows that NIP was significantly associated withtypes of mental illness, types of anti-psychotics, illness relapse, comorbid medical illness, medication for NIMD, comorbid medical illness, perceived stigma and current use of khat.

The odds of developing NIP among clients who had a diagnosis of Bipolar disorder were 3times **(AOR = 3.13**,** 95%CI:1.05**,** 9.25)**higher as compared to clients with a diagnosis of major depressive disorder. Clients having comorbid medical illness were 3 times **(AOR = 3.16**,** 95%CI: 1.32**,** 7.54)** more likely to develop NIP as compared to those clients without comorbid medical illness.

The odds of developing NIP among clients who had illness relapse were 2 times **(AOR = 2.11**,** 95%CI:1.26**,** 3.54)** higher as compared to married clients. Clients those taking Haloperidol were 2 times **(AOR = 2.30**,** 95%CI: 1.14**,** 4.65)**,and others were 2.86 times **(AOR = 2.86**,** 95%CI: 1.08**,** 7.55)** more likely to develop NIP as compared to those were taking Chlorpromazine. Clients those taking anticholinergic medication were 2 times **(AOR = 2.15**,** 95%CI: 1.27**,** 3.65**) more likely to develop NIP as compared to their counterpart. Clients who have perceived stigma were 3.47times **(AOR = 3.47**,** 95%CI: 2.03**,** 5.93)** more likely to develop NIP than clients without perceived stigma. The odds of developing NIP among clients who current khat users were 8 times **(AOR = 8.18**,** 95%CI: 2.01**,** 33.25)** more likely than clients who hadn’t taking khat (Table 4).

**Table 4:** Factors associated with antipsychotic induced parkinsonism (Bivariate analyses and multivariate analysis) among patients taking antipsychotics at general and referral public hospitals in Tigray, Ethiopia, 2019/2020 (*N* = 817)

Variable	Category	Parkinsonism		COR	AOR
		Yes	No		
Educational status	Unable to read and write	23	198	0.75(0.41,1.40)	
	Able to read and write	7	169	0.27(0.11,0.64)*	
	1–8	16	121	0.86(0.43,1.70)	
	9–12	12	98	0.79(0.38,1.67)	
	College and above	23	150	Ref	
Types of mental disorder	schizophrenia	51	485	1.01(0.46,2.21)	1.04(0.45,2.41)
	Other psychotic	12	138	0.83(0.32,2.13)	0.98(0.36,2.71)
	Bipolar	10	36	2.67(0.97,7.34)	3.13(1.05,9.25)*
	Major depressive	8	77	Ref	Ref
Types of anti-psychotics	Chlorpromazine	14	276	Ref	Ref
	Risperidone	16	144	2.19(1.04,4.61)*	1.78(0.79,3.97)
	haloperidol	43	273	3.11(1.66,5.81)***	2.30(1.14,4.65)*
	Others	8	43	3.66(1.45,9.26)**	2.86(1.08,7.55)*
Illness relapse	Yes	41	205	2.65(1.66,4.22)***	2.11(1.26,3.54)**
	No	4o	531	Ref	Ref
Comorbid medical illness	Yes	9	26	3.41(1.54,7.56)**	3.16(1.32,7.54)**
	No	72	710	Ref	Ref
Medication use for NIMD	Yes	40	169	3.29(2.06,5.26)***	2.15(1.27,3.65)**
	No	41	567	Ref	Ref
Drug none adherence	Yes	48	264	2.60(1.62,4.15)***	
	No	33	472	Ref	
Perceived stigma	Yes	58	281	4.08(2.46, 6.77)***	3.47(2.03,5.93)***
	No	23	455	Ref	Ref
Ever use of khat	Yes	15	75	2.00(1.08,3.68)*	1.19(0.54,2.62)
	No	66	661	Ref	Ref
Current use of khat	Yes	4	6	6.32(1.74,22.88)*	8.18(2.01,33.25)**
	No	77	730	Ref	Ref
Ever use of tobacco	Yes	9	26	3.41(1.54,7.56)*	1.52(0.47,4.91)
	No	72	710	Ref	Ref

Note: COR = Crude Odds Ratio; AOR = Adjusted Odds Ratio. Ref. = Reference category

* *p* < 0.05; ** *p* < 0.01; *** *p* < 0.001. Hosmer and Lemeshow test P-value = 0.064

### Clinical global impression of severity of antipsychotic induced extrapyramidal symptoms

The majority of participants (91.8%) did not exhibit dyskinesia. Mild symptoms were observed in 7.5%, moderate symptoms in 0.7%, and no cases of severe dyskinesia were reported.90.7% of participants showed no signs of parkinsonism. Mild parkinsonism was observed in 7.7%, moderate in 0.4%, and severe in 1.2% of cases.92.4% of individuals did not experience dystonia. Mild symptoms were present in 6.7%, moderate in 0.6%, and severe dystonia in only 0.2% of cases. This was the most frequently reported DIMD among the four. While 84.2% had no symptoms, a noTable 14.6% experienced mild akathisia. Moderate and severe cases were less common, affecting 1.0% and 0.2% of participants, respectively (Table 5).

**Table 5:** Clinical global impression of severity of antipsychotic-induced movement disorders among study participants at general and referral public hospitals in Tigray, Ethiopia (2024) (*n* = 817)

Severity Level	Dyskinesia, *n* (%)	Parkinsonism, *n* (%)	Dystonia, *n* (%)	Akathisia, *n* (%)
None	750 (91.8)	741 (90.7)	755 (92.4)	688 (84.2)
Mild	61 (7.5)	63 (7.7)	55 (6.7)	119 (14.6)
Moderate	6 (0.7)	3 (0.4)	5 (0.6)	8 (1.0)
Severe	0 (0.0)	10 (1.2)	2 (0.2)	2 (0.2)

## Discussion

This study aimed to evaluate the prevalence and associated factors of both acute and chronic extrapyramidal symptoms. The observed prevalence rates—9.9% for Parkinsonism, 6.9% for Akathisia, 5% for dystonia, and 2.4% for tardive dyskinesia—align closely with data from research conducted across 14 European countries [20],as well as studies in Europe. The study’s findings on the prevalence of various movement disorders are consistent with other international research, highlighting a similar trend in multiple regions, including Europe and Korea [18, 21]. The observed similarities in the prevalence of Parkinsonism, Akathisia, dystonia, and Tardive dyskinesia across different geographic regions may indicate the influence of common risk factors. These factors could include exposure to certain medications—particularly the use of second-generation antipsychotics and genetic predispositions [22].

In this study, the prevalence of akathisia is lower than that reported in previous studies from Ethiopia (21.2%–28.6%) [23], Estonia (31.3%) [24], the Netherlands (28.4%) [25], the UK (27%) [17], and a systematic review conducted in the USA (25.9%) [26]. This discrepancy may be attributed to differences in study setting, design, duration, and sample size. However, the rate observed here is slightly higher than that found in a study from southern Taiwan (2.4%) [27], suggesting possible regional or methodological variations. Similarly, the prevalence of drug-induced Parkinsonism in this study (9.9%) is also lower than those reported in studies from Ethiopia (20%–46.4%) [23, 25] South Taiwan (12.5%) [27], Estonia (23.2%) [24], the Netherlands (56.2%) [25], the UK (47%) [17], and a study in Filipino (20% to 53%) [28].These differences may be due to variability in diagnostic approaches, antipsychotic prescribing patterns, and patient characteristics across study populations.

The prevalence of tardive dyskinesia (2.4%) in this study is notably lower than findings from Estonia (32.3%) [24],the Netherlands (28.4%) [25], the UK (24%) [17], a European study (11%) [20], a systematic review from the USA (9.4%), a retrospective study (32.4%),in Taiwan (42.4–51.3%) [29], and others reporting rates between 13% and 20%. These differences may reflect variations in the type of antipsychotics used, especially the higher reliance on second-generation antipsychotics in this setting, the route of administration (oral vs. injectable), study design, and follow-up period. Interestingly, the result aligns with a study from Taiwan [27], which also reported a prevalence of 2.4%, possibly due to similarities in treatment practices, antipsychotic profiles, or monitoring strategies. Regarding tardive dystonia, the prevalence in this study (5%) is consistent with rates reported in Ethiopia (3.4%) [23], the Netherlands (5.7%) [25], and a prospective European study (3.3%) [20]. This similarity may be explained by comparable diagnostic standards, use of similar medication classes, and consistent methodologies in symptom detection. However, the rate is substantially lower than those found in studies from South Taiwan (21.1%) [27] and the UK (13%) [17]. This gap could be influenced by differences in diagnostic criteria, antipsychotic type and dosage, treatment duration, and genetic or environmental factors. Additionally, more rigorous monitoring or use of specific assessment tools in higher-prevalence studies may partly account for the discrepancies. Moreover, variations in diagnostic criteria or awareness of these disorders may also influence the differences in reported prevalence [23, 30].

The odds of developing parkinsonism were 3 times higher in clients diagnosed with Bipolar Disorder compared to those with Major Depressive Disorder (MDD) (AOR = 3.13, 95% CI: 1.05, 9.25). Infact bipolar patients are more prone to Parkinsonism and other EPS compared to those with MDD due to the nature of their condition and the medications used to manage it [31, 32]. This is likely due to the different pharmacological approaches used for treating these disorders, with Bipolar Disorder often requiring more aggressive antipsychotic treatments that are associated with higher risks of movement disorders [33]. This finding is supported with a previous study on the pharmacological treatment of mania across 14 European countries, which found a higher rate of EPS linked to the use of antipsychotic medications [20].Additionally, the finding that individuals with comorbid medical conditions are three times more likely to develop Parkinsonism is significant. The adjusted odds ratio (AOR) of 3.16, with a 95% confidence interval (CI) of 1.32 to 7.54, indicates a statistically significant association between comorbidities and Parkinsonism risk. Several factors may contribute to this increased risk. Patients with comorbid conditions often take a wider variety of medications, some of which can have side effects leading to movement disorders. Furthermore, the physiological and neurological stress of managing multiple health issues can exacerbate the likelihood of developing Parkinsonism. The specific nature of the comorbidities is also important; chronic conditions like diabetes and hypertension can negatively affect neurological health, potentially leading to changes in the brain that make individuals more susceptible to movement disorders [34].Research from Thailand and Germany strengthens the argument that comorbid conditions and the use of certain medications play a critical role in the development of Parkinsonism and other EPS [35, 36].

Clients taking haloperidol were found to be twice as likely to develop neuroleptic-induced Parkinsonism compared to those taking chlorpromazine, with an adjusted odds ratio (AOR) of 2.30 (95% confidence interval [CI]: 1.14, 4.65). This is supported **by** recent findings **indicating** that clients taking Haloperidol are at a notably higher risk of developing neuroleptic induced Parkinsonism, being **two** times more likely compared to those treated with Chlorpromazine. In addition to Haloperidol, clients on other antipsychotic medications also face an increased likelihood of developing neuroleptic induced parkinsonism, with an adjusted odds ratio (AOR) of 2.86 (95% CI: 1.08 to 7.55). This is supported by a study conducted in Ethiopia and Canada, which found that individuals taking typical antipsychotics were significantly associated with the development of Parkinsonism [23, 37].

Clients who have perceived stigma were 3.47 times (AOR = 3.47, 95%CI: 2.03, 5.93) more likely to develop neuroleptic induced parkinsonism than clients without perceived stigma and the odds of developing neuroleptic induced parkinsonism among clients who are current khat users were eight times (AOR = 8.18, 95% CI: 2.01 to 33.25) more likely than those who had not taken khat. This is highlighted by the compelling evidence that the stigma surrounding substance users is strongly linked to the prevalence of hypokinetic parkinsonism [38, 39].

The odds of developing Parkinsonism are two times higher in clients who have experienced an illness relapse compared to those who have not (AOR = 2.11, 95% CI: 1.26, 3.54). This increased risk underscores the significant impact that psychiatric relapses can have on the onset of movement disorders, particularly in individuals receiving antipsychotic treatment. Relapse in mental health conditions often requires changes or intensification of treatment, which can increase the risk of side effects, including neuroleptic induced Parkinsonism. The exacerbation of symptoms due to a relapse may further increase this risk, creating a concerning cycle where psychiatric instability leads to neurological complications. A growing of Research has shown that patients with antipsychotic disorders may have an inherent predisposition to developing side effects, with relapse associated with a 17% increase in the occurrence of Extrapyramidal symptoms [40].

On the other side also, the study’s findings indicated that individuals with a history of illness relapse have significantly higher odds of developing akathisia compared to those without such a history. One interpretation of these results is that fluctuations in psychiatric health, such as relapses, might contribute to increased sensitivity to the side effects of medications or dysregulation of neurotransmitter systems involved in both psychiatric symptoms and movement disorders. When patients experience a relapse, they may be exposed to higher doses or different classes of medications to stabilize their condition, potentially heightening the risk of developing akathisia [41]. Additionally, the development of akathisia in patients who have experienced relapses may also be reflective of the overall stress and instability associated with these episodes. The psychological distress that accompanies a relapse can impact a patient’s overall well-being, potentially increasing the likelihood of experiencing side effects from their medications [42].

Clients receiving haloperidol were 2.64 times more likely to develop akathisia, while those taking other types of antipsychotics faced a 3.42 times increased risk compared to individuals prescribed chlorpromazine. Additionally, clients on anticholinergic medications were 2.06 times more likely to develop akathisia compared to their counterparts. Furthermore, literature cited in Medline reports that medications such as haloperidol, ziprasidone, risperidone, aripiprazole, and quetiapine are significantly associated with an increased risk of developing akathisia, while anticholinergic use has been identified as a factor associated with akathisia rather than a direct causal agent [42]. The odds of developing Akathisia among clients who recently use khat were 6.99 times more likely to develop akathisia than clients who had not taken khat. This is supported by a comparative study involving patients with dual diagnoses of schizophrenia and substance use disorders, which found that akathisia was significantly associated with substance use disorders in those taking antipsychotic medications [35, 43].

Moreover, this study revealed that dystonia was significantly associated with factors such as marital status, the use of anticholinergic medications, perceived stigma, and tobacco use. Divorced clients faced a 3.98 times greater likelihood of developing dystonia (AOR = 3.98, 95% CI: 1.67–9.49) compared to married individuals. This heightened risk may be linked to the alcohol and cocaine use prevalent among many divorced individuals, which can increase the chances of experiencing acute dystonia reactions [44]. Clients who were on anticholinergic medication had a 3.41 times higher likelihood of developing dystonia (AOR = 3.41, 95% CI: 1.76–6.59) compared to those not using anticholinergics [45].

The findings indicate that clients currently using khat are 11 times more likely to develop dystonia compared to those who do not use it, with an adjusted odds ratio (AOR) of 11.02 and a 95% confidence interval (CI) of 1.67 to 72.74. This significant association raises important concerns regarding the neurological effects of khat and underscores the necessity of monitoring potential adverse reactions among users. Khat, a stimulant containing cathinone, has been associated with various health issues beyond its psychoactive effects. The link between khat use and dystonia suggests that the substance may have direct neurological impacts, possibly affecting dopaminergic pathways or other neurotransmitter systems involved in movement control. This correlation highlights the need for healthcare providers to remain vigilant when treating individuals who use khat, as they may be at increased risk for movement disorders [46, 47].

This study further highlights that clients who experienced perceived stigma were 2.8 times more likely to develop dystonia compared to those who did not perceive any stigma, with an adjusted odds ratio (AOR) of 2.8 and a 95% confidence interval (CI) of 1.40 to 5.59.These findings indicate a significant association between the psychological burden of stigma and the risk of developing dystonia. Perceived stigma can lead to feelings of shame, social isolation, and increased stress, all of which can have detrimental effects on both mental and physical health. For individuals already vulnerable to neurological conditions, the heightened stress associated with stigma may exacerbate or precipitate the development of movement disorders [48]. The findings from the recent study indicate that stigma against generalized dystonia is widespread across various communities, with a notable prevalence rate of 33.00%. This level of stigma is comparable to that observed for epilepsy but lower than the stigma associated with schizophrenia [49]. This is also supported by previous research indicating that clients who perceive stigma are 3.73 times more likely to develop Extrapyramidal symptoms compared to those who do not experience such stigma. Clients who face stigma related to their mental health or treatment may encounter heightened emotional and psychological stress, which can exacerbate existing conditions or contribute to the development of movement disorders. Individuals who feel stigmatized may be less likely to adhere to prescribed treatments or may avoid seeking help altogether, which can lead to worse health outcomes, including the development of drug-induced movement disorders. Additionally, stigma may result in social isolation, increased anxiety, or depression—factors that have been shown to heighten vulnerability to various health conditions, including movement disorders [50].

The present study adds to the limited evidence on the prevalence and associated factors of extrapyramidal side effects (EPSEs) among psychiatric patients in Ethiopia, particularly in the Tigray region. Previous studies have mainly focused on small-scale institutional assessments, with little data from routine hospital settings in resource-limited contexts. Our findings therefore address a significant research gap by providing population-based estimates of EPSE prevalence and identifying demographic, clinical, and medication-related predictors in real-world treatment environments. This contributes to a more contextual understanding of the burden and determinants of EPSEs, which is critical for improving psychotropic medication monitoring and management practices in similar settings.

Despite its contributions, this study has several limitations. The cross-sectional design restricts the ability to establish temporality or causality between exposure variables (e.g., type or duration of antipsychotic use) and the occurrence of EPSEs. Consequently, it is unclear whether the associated factors identified preceded or resulted from the side effects observed. Furthermore, this design does not permit the assessment of treatment changes, dosage adjustments, or symptom progression over time. The directionality of some associations—such as between medication adherence and EPSE occurrence—also remains uncertain. Future longitudinal and interventional studies are recommended to confirm these associations and explore causative pathways.

## Conclusions

This study found that the prevalence of antipsychotic-induced Extrapyramidal symptoms (EPS) among patients in follow-up care was lower than previously reported. Several factors were associated with EPS, reflecting the complex interplay between medication type, patient characteristics, and social variables. Parkinsonism and akathisia were more common among patients exposed to certain antipsychotic medications, those with illness relapse, comorbid medical conditions, use of anticholinergic medications, perceived stigma, or current khat consumption. Parkinsonism appeared more frequently in patients with specific mental health disorders, while dystonia was potentially associated with marital status, anticholinergic use, perceived stigma, and tobacco consumption. Tardive dyskinesia was observed more often in patients with lower educational status and suboptimal medication adherence. These findings highlight the importance of monitoring EPS in clinical practice, while further research is needed to confirm these associations in broader populations.

## Originality and contribution

This study makes a significant and unique contribution by featuring the largest sample size to date in the country, enhancing the reliability and generalizability of its findings. Conducted across five hospitals in a previously understudied, conflict-affected region, it fills a critical gap in the literature and offers new insights into the challenges faced in post-conflict settings. By expanding both geographic and methodological scope, it challenges existing perceptions, broadens knowledge, and has the potential to influence clinical practice, inform targeted interventions, and guide future research in similar contexts.

## Recommendations

Based on the study findings, healthcare providers should routinely monitor and screen patients during follow-up care for both acute and chronic Extrapyramidal symptoms using standardized screening tools such as the Extrapyramidal Symptom Rating Scale. Providers should take proactive steps to address stigma and acknowledge the psychological burden it places on patients. Furthermore, effective management of relapses is essential, along with a comprehensive evaluation of each patient’s overall medical status, including any comorbid conditions that may increase the risk of adverse effects. Treatment approaches for Extrapyramidal symptoms should be personalized, potentially requiring adjustments to the type of antipsychotic medication prescribed.

## Limitations and future directions

It is important to acknowledge that the cross-sectional study design limits the ability to establish causal relationships between identified risk factors and EPS. Longitudinal studies are necessary to clarify temporal dynamics and causality. Additionally, future research should explore intervention strategies to reduce EPS, assess long-term outcomes, and investigate underlying biological mechanisms, thereby providing a more comprehensive understanding and enhancing clinical management of these symptoms. This study did not fully control for the use of concomitant psychotropic medications in patients receiving antipsychotics. The concurrent use of such medications may have acted as a potential confounding factor, influencing the occurrence and severity of extrapyramidal symptoms. Therefore, the findings should be interpreted with caution in light of this limitation.

Another limitation of this study is that the concomitant use of other substances (such as alcohol, khat, and tobacco) was not fully controlled. The concurrent use of these substances may have introduced confounding effects, potentially influencing the occurrence and severity of extrapyramidal symptoms and thereby affecting the generalizability of the findings.

## Data Availability

The datasets used and/or analyzed during the current study are available from the corresponding author on reasonable request or interested qualified researchers can access the data by requesting the research review committee of, college of health Sciences via [hadgugerensea2015@gmail.com].

## Abbreviations

AIMS: Abnormal Involuntary Movement Disorder Scale
BARS: Barnes Akathisia Rating Scale
BPD: Bipolar Disorder
CGI-S: Clinical Global Impression of Severity Scale
DDD: Defined Daily Dose
DSM: Diagnostic and Statistical Manual of American Psychiatric Association
EPS: Extra pyramidal side-effect
ESRS: Extra Pyramidal Symptom Rating Scale
FDA: Food and Drug Administration
FGAs: First-Generation Antipsychotics
IRB: Institutional Review Board
MMAS: Morisky Medication Adherence Scale
NIA: Neuroleptic-Induced Akathisia
NITD: Neuroleptic-Induced Tardive Dyskinesia
SGAs: Second-Generation Antipsychotics
SAS: Simpson Angus Scale
TD: Tardive Dyskinesia
UPDRS: Unified Parkinson Disease Rating Scale
